# Three Decades of Cannabis Research: What are the Obstacles?

**DOI:** 10.5041/RMMJ.10486

**Published:** 2022-10-27

**Authors:** Michael Dor

**Affiliations:** Former Senior Medical Cannabis Consultant, Medical Cannabis Unit, Israel Ministry of Health, Jerusalem, Israel. Ariel University

**Keywords:** Cannabidiol, cannabis obstacles, cannabis research, medical cannabis

During the past few years, thousands of articles have been published concerning medical cannabis usage. Unfortunately, most publications are case studies or small and poorly designed research projects. The obstacles impeding the use of medical cannabis and related research merit more in-depth examination if we are to understand the reasons behind this situation.

## LEGISLATIVE AND REGULATORY BARRIERS

In many countries, the fundamental obstacle to cannabis research is a result of antagonistic legislation; in some places, there are legal prohibitions on using cannabis for medical purposes.^1^ For several years, cannabis use was forbidden for medical treatment or research in the United States (USA).^2^,^3^ Although federal USA legislation is still antagonistic toward medical cannabis use and research, the number of states approving it continues to grow. Many other countries have changed their legislation over the last decade, so as to remove cannabidiol (CBD) from the list of narcotics.^4^

Bureaucracy has been influential in delaying new laws and regulations facilitating cannabis research. In Israel, the process has been slow; cannabis legislation has been awaiting approval by the Knesset for more than ten years and will not be finalized any time soon due to current political uncertainty.

## NON-UNIFORMITY IN THE PRODUCTION PROCESS

Cannabis is a highly complex plant composed of about 600 different cannabinoids, 60 flavonoids, and 60 terpenes. Differences in genetic origin or growing conditions (temperature, moisture, type of soil, or light) lead to different compositions of the final drug produced.^5^ Uniformity of cannabis medication production is complicated, and manufacture according to good manufacturing practice (GMP) guidelines is difficult and costly. Reliable and reproducible production and manufacture are essential to the conduct of valid research that complies with the requirements of Research Ethics Committees.

## PREJUDICE

The active role played by interested parties in the media, who either condemn or promote the use of cannabis for medical or social purposes, has created a highly complex situation. In most of the world, cannabis use for social purposes remains controversial.^6^ Some patients fear using it because of the stigma cannabis carries as a “forbidden substance.” Many physicians are concerned that being involved with cannabis treatment might hurt their professional reputation. At the other extreme are those influenced by the intensive promotion of cannabis and expect it to cure every sickness—a kind of “panacea.” The increasing number of cannabis-treated patients, many of whom are also physicians, is gradually changing the way physicians view this treatment. If implemented, quality research would play an essential role in how cannabis is prescribed.

## LACK OF RELIABLE INFORMATION

Most medical professionals lack knowledge concerning cannabis treatment, making them unable to provide reliable information to their patients. Compounding the situation is the plethora of not necessarily accurate information about “medical” cannabis via social media and other non-professional sources.^7^,^8^ To counter this, the curriculum of medical schools should cover and discuss cannabis treatment in general, and particularly for relevant specializations such as pain and cancer.

## BUDGETARY ISSUES

Medical research is costly. For many years, pharmaceutical companies were reluctant to invest money in cannabis research projects.^7^ However, recent years have seen a change of attitude by many large companies, which are starting to finance medical cannabis studies.

## LEGAL PROTECTION (INSURANCE)

Allowing medical use of a substance not yet approved as a medication raises both ethical and legal questions. As long as the treatment outcomes are questionable, accountability may be problematic. From an ethical perspective, physicians would be more motivated to prescribe medical cannabis if there was more research that demonstrated positive clinical outcomes.

The legal issues also depend on local circumstances. In Israel, the legality of medical cannabis is covered by official guidelines published by the Ministry of Health.^9^ These guidelines provide a legal umbrella allowing research, assuming the studies are performed and authorized in accordance with the local Research Ethics Committee and the appropriate committee within the Ministry of Health.

## INSPIRATION FOR PROGRESS

Israel is proud to have among its ranks the foremost influential cannabis researcher worldwide, Professor Raphael Mechoulam. His research, which started more than 30 years ago, remains a cornerstone of cannabis research.^10^ Despite social, legal, and prejudicial obstacles, his pioneering work and international reputation enable Israeli researchers to pursue studies into cannabis for medical use. The October 2022 issue of *Rambam Maimonides Medical Journal* includes a paper that represents one example of such studies.

Dror Robinson and colleagues compared the sublingual administration of cannabis oil extracts to inhaled cannabis in patients suffering from chronic low back pain.^11^ Their study represents a real-world situation in which the exact cannabis ingredients were variable. Few studies have compared different forms of cannabis administration. As the authors explain, the conclusion of their study is equivocated by the inability to evaluate similar compounds. Still, Robinson et al.’s evaluation of two forms of cannabis administration in patients with a commonly encountered chronic pain syndrome should be an inspiration for further medical cannabis research into the effectiveness of different administration routes.

## Abbreviations

CBN: cannabidiol
USA: United States of America.
